# Symbiont-driven sulfur crystal formation in a thiotrophic symbiosis from deep-sea hydrocarbon seeps

**DOI:** 10.1111/1758-2229.12149

**Published:** 2014-03-03

**Authors:** Irmgard Eichinger, Stephan Schmitz-Esser, Markus Schmid, Charles R Fisher, Monika Bright

**Affiliations:** 1 Department of Limnology and Oceanography, Faculty of Life Sciences, University of ViennaAlthanstr. 14, 1090, Vienna, Austria; 2 Department of Microbial Ecology, Faculty of Life Sciences, University of ViennaAlthanstr. 14, 1090, Vienna, Austria; 3 Institute for Milk Hygiene, University of Veterinary Medicine ViennaVeterinärplatz 1, 1210, Vienna, Austria; 4 Department of Biology, Pennsylvania State University208 Mueller Laboratory, University Park, Schuylkill, PA, 16802, USA

## Abstract

The siboglinid tubeworm *Sclerolinum contortum* symbiosis inhabits sulfidic sediments at deep-sea hydrocarbon seeps in the Gulf of Mexico. A single symbiont phylotype in the symbiont-housing organ is inferred from phylogenetic analyses of the 16S ribosomal ribonucleic acid (16S rRNA) gene and fluorescent in situ hybridization. The phylotype we studied here, and a previous study from an arctic hydrocarbon seep population, reveal identical 16S rRNA symbiont gene sequences. While sulfide is apparently the energy source for the symbionts (and ultimately the gutless host), both partners also have to cope with its toxicity. This study demonstrates abundant large sulfur crystals restricted to the trophosome area. Based on Raman microspectroscopy and energy dispersive X-ray analysis, these crystals have the same S8 sulfur configuration as the recently described small sulfur vesicles formed in the symbionts. The crystals reside adjacent to the symbionts in the trophosome. This suggests that their formation is either extra- or intracellular in symbionts. We propose that formation of these crystals provides both energy-storage compounds for the symbionts and serves the symbiosis by removing excess toxic sulfide from host tissues. This symbiont-mediated sulfide detoxification may have been crucial for the establishment of thiotrophic symbiosis and continues to remain an important function of the symbionts.

## Introduction

Hydrogen sulfide is an energy-rich source for chemolithoautotrophic, sulfur-oxidizing bacteria, but it is also highly toxic to aerobic organisms because of its inhibition of the respiratory enzyme cytochrome c oxidase at even nanomolecular concentrations (National Research Council, Division of Medical Science, subcommittee on Hydrogen Sulfide, 1979). Nevertheless, many animals and protists inhabit sulfidic marine environments such as hydrothermal vents, hydrocarbon seeps, whale and wood falls, sewage outfalls, mangrove swamps and other reduced sediments, and some of them live in symbiosis with thiotrophic bacteria (Dubilier *et al.*, 2008). Such hosts may help provide their symbionts with reduced sulfur species and oxygen for chemoautotrophy but at the same time need to avoid sulfide poisoning.

The siboglinid annelid tubeworms in the genus *Sclerolinum*, which are closely related to the better known hydrothermal vent and cold seep vestimentiferan tubeworms, live in reducing marine sediments with access to sulfide from interstitial waters and oxygen in the overlying epibenthic water. One species, *Sclerolinum contortum* (Smirnov, 2000), has been reported from hydrocarbon seeps in the Arctic Sea (Lösekann *et al*., 2008; Lazar *et al.*, 2010) and in the Gulf of Mexico (Eichinger *et al.*, 2013), as well as from an arctic hydrothermal vent field (Pedersen *et al.*, 2010).

Lacking a digestive system as adults, siboglinids live in obligate symbiosis with intracellular bacteria belonging to at least three distinct clades of *Gammaproteobacteria* (Dubilier *et al.*, 2008). Thiotrophic symbionts of vestimentiferans and *S. contortum* are divergent from both the thiotrophic and methanotrophic bacteria associated with frenulates as well as the heterotrophic bacteria of *Osedax* (McMullin *et al.*, 2003; Goffredi *et al.*, 2005; Lösekann *et al*., 2008; Thornhill *et al.*, 2008).

The bacterial symbionts are encased in membrane-bound symbiosomes within host cells termed bacteriocytes in a highly vascularized organ, the trophosome (Cavanaugh *et al.*, 1981; Southward, 1982; Eichinger *et al.*, 2011; Katz *et al.*, 2011). Symbionts of *S. contortum* were suggested to exhibit a cell cycle directed from anterior to posterior within the trophosome located in the worm's trunk region. Anteriorly, a small proliferating bacterial stem population is housed in a few bacteriocytes. In the posterior trophosome region, however, the bacteriocytes that fill the whole body cavity are full of symbionts containing membrane-bound S8 sulfur vesicles. Bacteria in various stages of degradation are scattered among intact symbionts in the posterior trophosome (Eichinger *et al.*, 2011).

In the much better studied sister taxon of *Sclerolinum*, the vestimentiferans, carbon dioxide is transported freely dissolved in the blood (Goffredi *et al.*, 1997), whereas oxygen and sulfide bind simultaneously and reversibly to giant haemoglobin molecules in the blood (Arp and Childress, 1983; Arp *et al*., 1985; 1987,). These carriers transport and release sulfide to the symbionts while simultaneously suppressing spontaneous oxidation of sulfide and protecting the host tissue and the symbionts from sulfide toxicity by keeping free sulfide concentrations low (Fisher *et al.*, 1988). *Sclerolinum contortum* has haemoglobins with a similar structure to those of vestimentiferans (Meunier *et al.*, 2010) but a sulfide-binding ability has not been demonstrated.

Since the discovery of the symbiotic lifestyle of Siboglinidae much emphasis was put on the nutritional relationship (Felbeck, 1981; Southward *et al*., 1981; 1986; Cavanaugh, 1983; Felbeck and Jarchow, 1998; Bright *et al.*, 2000). However, here we investigate the possibility that the symbionts protect their hosts from harmful effects of sulfide by oxidizing it to a non-toxic form they accumulate, a potentially crucial initial adaptive trait for establishment of such a symbiosis (Vismann, 1990; 1991a,). In this study, the symbiotic bacteria of *S. contortum* from the hydrocarbon seeps of the Gulf of Mexico were identified based on 16S rRNA gene sequencing, comparative sequence analyses and specific fluorescence in situ hybridization (FISH). We demonstrate the presence of large sulfur crystals within the trophosome, restricted to posterior areas of the animals that are exposed to sulfide in the sediments, using high-pressure freezing and freeze substitution for light microscopy (LM), scanning electron microscopy, Raman microspectroscopy and energy dispersive X-ray analysis (EDX). We further provide evidence from electron micrographs that symbiotic bacteria produce extracellular sulfur deposits resulting in gigantic crystals. We propose that the endosymbionts play a major role in sulfide detoxification by producing non-toxic sulfur crystals from excess hydrogen sulfide that enters the worm, thus enabling the host to reside in an environment with an abundant energy source for its symbionts and reduced competition and predation because of its toxicity to most other fauna.

## Results and discussion

We discovered three hydrocarbon seep sites at depths between 2000 and 2700 m in the Gulf of Mexico with dense populations of *Sclerolinum contortum* tubeworms (In Bureau of Ocean Energy Management lease blocks WR269, AC818, AC601). Most of the tube was straight and buried in mud, but the anterior end was curled and extended above the seafloor (Eichinger *et al.*, 2013).

### Symbiont identification

A single bacterial phylotype was identified in each of nine clones from trophosome tissue of one *S. contortum* specimen from WR 269 based on 16S rRNA gene sequence (Supporting information). The software pintail (Ashelford *et al.*, 2005) indicated that the obtained sequences were not chimeric. The consensus sequence (GenBank accession number HE614013) was 100% identical to the *S. contortum* endosymbiont from the arctic Haakon Mosby Mud Volcano (Lösekann *et al*., 2008). Similarly, the host *S. contortum* from the Gulf of Mexico and the Northeast Atlantic show no genetic diversity at the 16S rRNA gene level (Eichinger *et al.*, 2013). These results indicate a specific and conserved association between *S. contortum* and its symbionts and a wide geographic distribution of this association.

**Fig 1:**
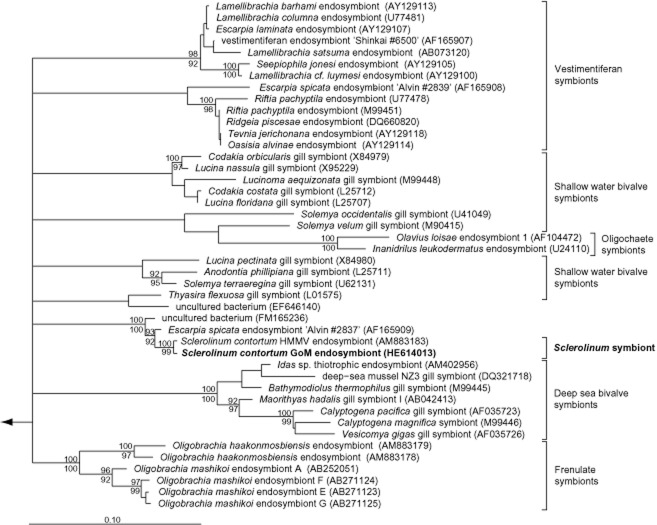
Phylogeny of the endosymbiont of *S. contortum* from the Gulf of Mexico and other gammaproteobacterial symbionts based on 16S rRNA gene sequences and ARB analyses (Ludwig *et al.*, 2004). A consensus tree calculated by the raxml maximum-likelihood algorithm implemented in ARB is shown. A filter considering only positions, which are conserved in at least 50% of all gammaproteobacterial 16S rRNA sequences, was used for tree calculations. Maximum parsimony bootstrap values are depicted above the respective branches, raxml bootstrap values are shown below the respective branches; only bootstrap values above 90% are shown, GenBank accession numbers are given in parentheses. Alphaproteobacterial 16S rRNA sequences were used as out-group. The arrow points to the out-group, and the bar represents 10% estimated evolutionary distance. The sequence obtained in this study is highlighted in bold. GoM, Gulf of Mexico; HMMV, Haakon Mosby Mud Volcano.

**Fig 2:**
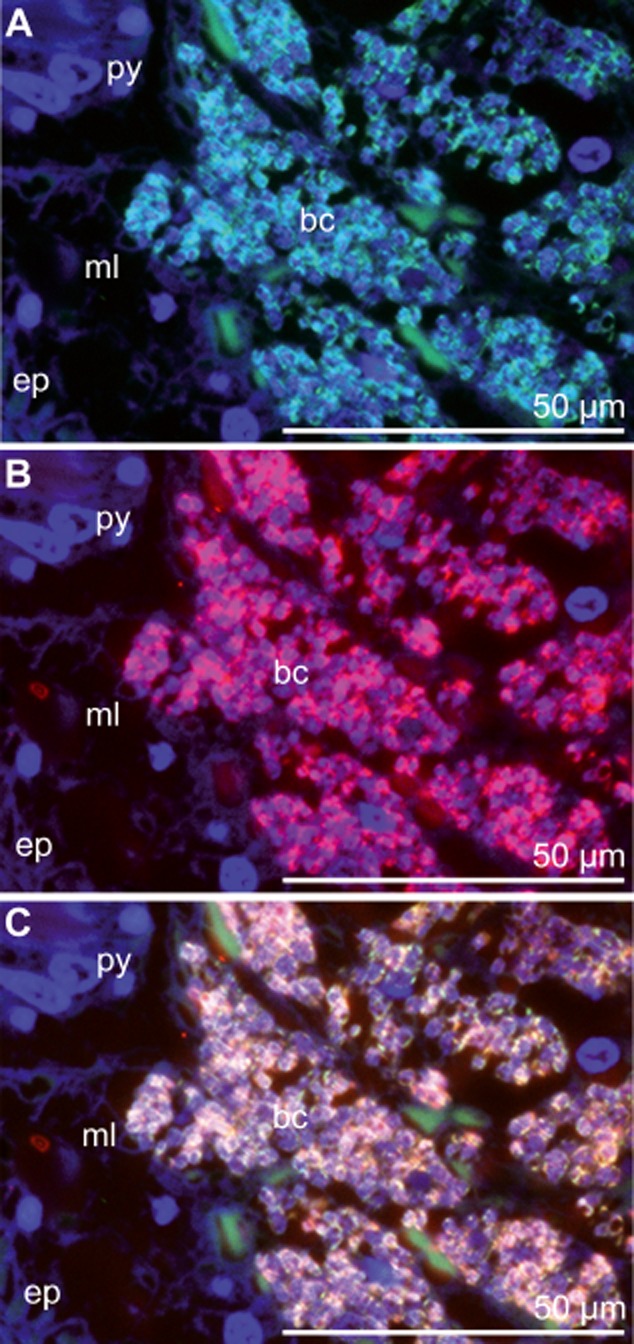
FISH micrographs of cross sections through the posterior trophosome region treated with an hierarchical probe set. 4′,6-diamidino-2-phenylindole (DAPI) was used as a counter stain (blue). A. Probe Gam42a in Fluos (green) targeting *Gammaproteobacteria*. B. Probe Scon −467 in Cy5 (red) specific for the *S. contortum* endosymbionts. C. Overlay. Bc, bacteriocyte; ep, epidermis; ml, muscle layer; py, pyriform gland.

The *S. contortum* symbiont sequences formed a stable clade with the endosymbiont of the vestimentiferan *Escarpia spicata* (98.5% similarity) from a Guaymas Basin vent and an uncultured bacterium (98% similarity) associated with tubes of the vestimentiferan *Lamellibrachia* sp. from cold seeps of the Mediterranean Sea (Fig. 1). Double FISH with a symbiont-specific oligonucleotide probe and general probes specific for most *Bacteria* and *Gammaproteobacteria*, respectively, confirmed the exclusive presence of the single symbiont 16S rRNA phylotype within the trophosome of four animals from two different locations (WR 269, AC 818) (Supporting information) (Fig. 2). The presence of the functional genes *cbbM* and *aprA* studied in the endosymbiont of the mud volcano population (Lösekann *et al*., 2008) indicate that this symbiont, like the symbiont of *E. spicata*, is a sulfur-oxidizing autotroph (Nelson and Fisher, 1995).

Although the symbionts of the sister taxon vestimentifera generally fall into well-defined clades congruent to higher level taxonomic phylogeny of the hosts, the symbiont phylogeny does not correspond with the vestimentiferan's at the species level. Rather the larval vestimentiferan host acquires the locally available free-living symbiotic bacterial strain associated with the specific regional ecosystem (Feldman *et al.*, 1997; Di Meo *et al.*, 2000; Nelson and Fisher, 2000; McMullin *et al.*, 2003; Vrijenhoek *et al.*, 2007). The fact that the *S. contortum* symbiont did not cluster with symbionts of cold seep vestimentiferans *Escarpia laminata* and *Lamellibrachia* sp, from the same sites (McMullin *et al.*, 2003; Roberts *et al.*, 2007), but instead is most closely affiliated with the endosymbiont found in the vestimentiferan *E. spicata* from a geographically distant hydrothermal vent in the Pacific Ocean (Di Meo *et al.*, 2000) suggests possible differences in the mode of symbiont transmission. Interestingly, *S. contortum* is also known from hydrothermal vents (Pedersen *et al.*, 2010), however genetic information on the symbiont from individuals at these sites is not available.

### Sulfur crystals within the trophosome

**Fig 3:**
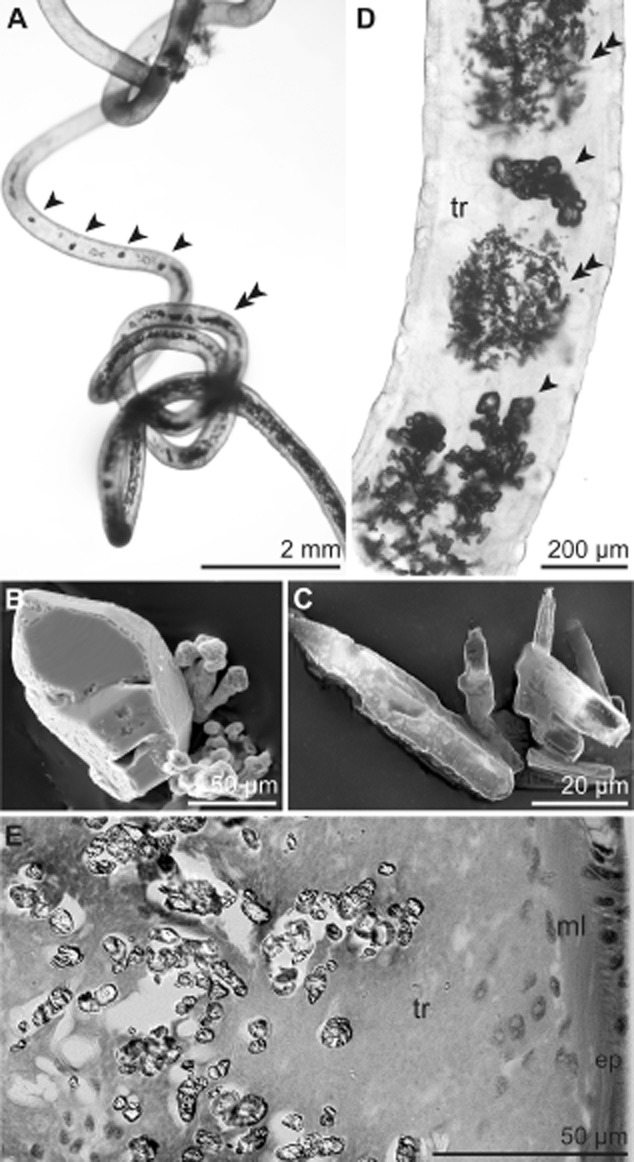
Crystals deposited in the *S. contortum* trophosome. A. Whole specimen within the tube viewed under a dissecting microscope containing orthorhombic (arrowhead) and needle-shaped crystals (double arrowhead). B–C. SEM of orthorhombic (B) and needle-shaped crystals (C). D. LM of whole mount of the posterior body region showing regions of densely packed needle-shaped crystals (double arrowhead) interspersed by orthorhombic ones (arrowhead). E. LM of high-pressure frozen and freeze-substituted sample of the posterior trophosome. Crystals are limited to the trophosomal tissue located in the body cavity of the trunk. Ep, epidermis; ml, muscle layer; tr, trophosome.

Five specimens fixed in 4% formaldehyde buffered with 0.1 mol l–1 phosphate-buffered saline (PBS), 5.4 to 8.6 cm in length, revealed abundant, giant crystals that were even visible through the worm's tube under the dissecting microscope (Fig. 3A). Two kinds of water insoluble crystals were detected: tightly packed needle-shaped crystals up to 50 μm in length interspersed with clumps of orthorhombic crystals up to 150 μm in length (Fig. 3B–D). LM of whole mounts indicated a restricted distribution of the crystals to the posterior trophosomal tissue, which is deeply buried in sulfidic mud in situ. Further examination of semithin sections of high-pressure frozen and freeze-substituted samples, infiltrated by Lowicryl HM20 resin (Supporting information) under the LM indicated the crystals were located in cavities between bacteriocytes filled with symbionts (Fig. 3E).

**Fig 4:**
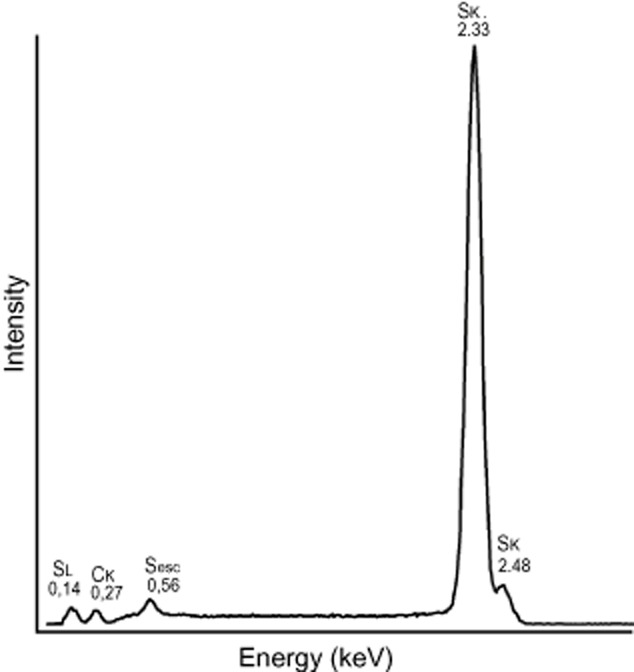
Representative EDX spectrum of isolated crystal with peaks at 0.14 keV, 0.56 keV, 2.33 keV and 2.48 keV characteristic for sulfur. A small carbon peak at 0.27 keV is due to carbon coating.

**Fig 5:**
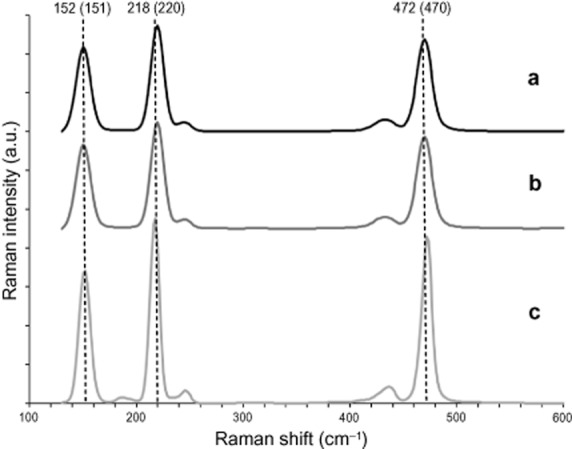
Raman microspectroscopy of the region between 100 cm^−1^ and 600 cm^−1^. A. Raman spectrum of orthorhombic crystals. B. Raman spectrum of needle-shaped crystals. C. Elemental S8 sulfur. Both crystal types showed very strong bands at about 470 cm^−1^ [sulfur–sulfur bond (S-S) stretching], 220 cm^−1^, and 151 cm^−1^ (both S8 bending), indicating that both crystal types consisted of rhombic S8 sulfur (Ward, 1968). Weaker bands at 245 cm^−1^ and 433 cm^−1^ also assignable to S8 sulfur (Trofimov *et al.*, 2009) could be observed, while the additional peak at 187 cm^−1^ visible in the S8 sulfur spectrum was below background level. The main bands indicative for measured S8 sulfur are indicated by vertical dotted lines. The numbers indicate the position of the bands in S8 sulfur in cm-1. The numbers in brackets indicate the wave numbers for the respective sulfur band in both crystal types.

EDX elemental analyses of isolated needle-shaped and orthorhombic crystals sputter coated with carbon confirmed the crystals were sulfur (Fig. 4). Raman microspectroscopic analysis of isolated and dried crystals with a spectral resolution of about 1.5 cm^−1^ (Eichinger *et al.*, 2011) indicated that that both crystal types were composed of rhombic S8 sulfur (Fig. 5).

**Fig 6:**
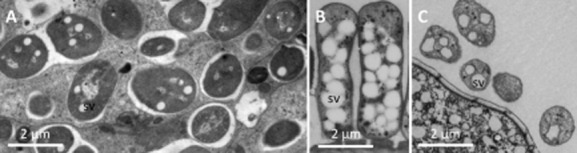
Bacterial elemental sulfur vesicles of different thiotrophic symbiosis. A–C. Symbionts of the vestimentiferan *Riftia pachyptila* (A), of the giant ciliate *Zoothamnium niveum* (B), and the stilbonematin nematode *Laxus oneistus* (C), all fixed similar to *S. contortum* as described in Eichinger and colleagues (2011). Sv, sulfur vesicle.

To our knowledge, the presence of such sulfur crystals has never been reported in a metazoan. Although we do not have micrographs of live animals, we can eliminate the possibility that these crystals are an artefact of the fixation protocol for three reasons: (i) the same distribution patterns of crystals in the trophosome are present in *S. contortum* fixed using a different [transmission electron microscopy (TEM)] fixative and buffer (see Eichinger *et al.*, 2013), (ii) previous work using the same fixative and buffer used in this study with various other thiotrophic ecto- and endosymbiotic bacteria associated with siboglinids tubeworms, stilbonematin nematodes or colonial ciliates (Fig. 6) never detected such crystals, although all these symbionts contain S8 sulfur vesicles (Pflugfelder *et al.*, 2005; Himmel *et al.*, 2009; Maurin *et al.*, 2010) and (iii) if these crystals were a fixation artefact resulting from transformation of S8 sulfur vesicles, the number of bacterial sulfur vesicles should be reduced or lacking in areas with large amounts of crystals. However, vesicles and crystals co-localized in large numbers in the posterior trophosome, while only a few vesicles and no crystals are present in the anterior trophosome. Therefore, an artificial transformation process from the amorphous to the crystalline state resulting from the specimen preparation protocol is highly unlikely.

### Symbiont-driven crystallization process

Several facts point to a symbiont origin of the sulfur crystals. Their occurrence is limited to the symbiont-housing organ and adjacent to symbionts, rather than distributed among symbiont-containing and symbiont-free host tissue. Furthermore, both bacterial sulfur vesicles and sulfur crystals have the same sulfur S8 configuration.

Combining static TEM micrographs into a reasonable process of crystal formation, we propose that the symbionts actively produce the large elemental sulfur crystals. We could not determine whether the crystals were directly deposited extracellularly, similar to the deposition process for amorphous extracellular sulfur vesicles in *Thiorhodospira sibirica* and other members of *Ectothiorhodospiraceae* (Bryantseva *et al.*, 1999; Dahl and Prange, 2006), or if they originate from the intercellular bacterial sulfur vesicles. The latter process never has been described but examination of the micrographs provides some evidence for this process.

**Fig 7:**
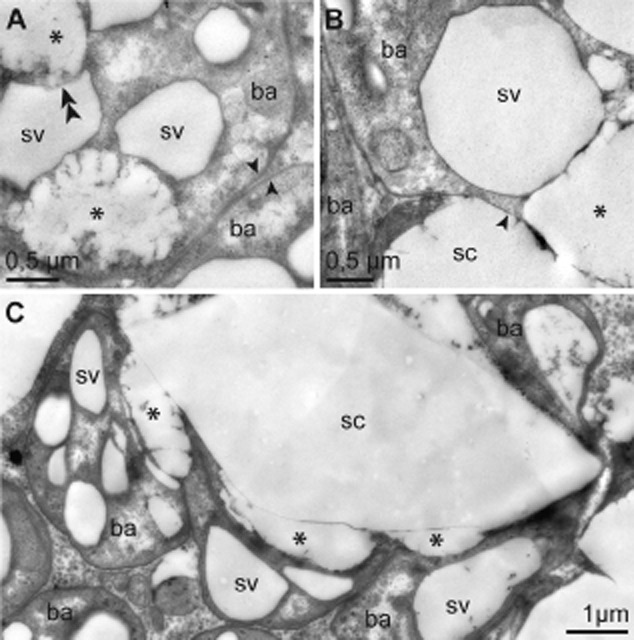
Sulfur crystal formation by the symbionts (TEM). A. Symbionts with intact cell wall (arrow), sulfur vesicles and remnants of sulfur vesicles caused by disintegration of vesicle membranes, indicated by double arrowhead. B. Detail of symbiont with partially disintegrated cell wall. Transition between intact and disintegrated cell wall is indicated by arrowhead. Remnant of sulfur vesicle next to sulfur crystal located within the bacteriocyte cytoplasm. C. Sulfur crystal completely surrounded by symbionts. Remnants of sulfur vesicles are located next to a crystal. Bacterial areas adjacent to a crystal lack a cell wall. Asterisk indicates remnant of bacterial sulfur vesicle. Note that neither the content of sulfur vesicles nor the crystals are actually still present in the TEM micrographs because of dissolution of sulfur during the dehydration and embedding process. Ba, bacterium; sc, sulfur crystal; sv, sulfur vesicle.

Viable symbionts, characterized by intact outer and cytoplasmic membranes and a moderately electron-dense cytoplasm containing glycogen and chromatin strands, contained small, membrane-bound, electron-translucent sulfur vesicles with S8 sulfur (Eichinger *et al.*, 2011). The first step in the transition for sulfur vesicles inside the symbionts to accumulation of crystals in host cell cytoplasm is the disintegration of the sulfur vesicle membrane. Such remnants of sulfur vesicles were present as diffuse electron-translucent patches within the symbiont's cytoplasm (Fig. 7A). Next, the remnants of the sulfur vesicles must pass through the symbiont's cell wall and the symbiosome membrane into the bacteriocyte cytoplasm (Fig. 7B). Here sulfur accumulates, which was visible as conspicuous, electron-translucent area often with straight edges typical of the crystals (Fig. 7C). Such areas were completely surrounded by symbionts with intact cell walls, except where they were adjacent to the crystals. Crystals are then found in bacteriocytes with an intact nucleus and cell membranes.

### Symbiont-driven sulfide detoxification

Elemental sulfur production combines two advantages to animals living in sulfidic environments: it is non-toxic and it does not require oxygen atoms for its formation from sulfide (Powell *et al.*, 1980). Elemental sulfur deposited in intracellular sulfur vesicles is known from many thiotrophic symbionts. Such vesicles, described as infoldings of the bacterial cytoplasmic membrane (Bright and Sorgo, 2003), were detected in the endosymbiont *Cand. Endoriftia persephone* of the vestimentiferan *Riftia pachyptila* (Pflugfelder *et al.*, 2005), a close relative of *S. contortum*. They also occur in the endosymbionts of gutless oligochaetes (Krieger *et al.*, 2000), the gutless platyhelminths (Gruber-Vodicka *et al.*, 2011) and several vesicomyid and lucinid clams (Vetter, 1985), as well as in the ectosymbionts of the giant ciliate *Zoothamnium niveum* (Maurin *et al.*, 2010). They have been proposed to be formed under oxygen limitation as an intermediate energy-storage product that may later be utilized when oxygen supply exceeds the rate of sulfide diffusion into the animal (Vetter, 1985). Evidence for this was found in *R. pachyptila* where the sulfur content of the trophosome changed in response to experimental changes in the relative levels of sulfide and oxygen exposure (Childress *et al.*, 1991).

Extracellular deposits of sulfur in vesicles (also termed globules) are known from many free-living bacteria of the families *Chlorobiaceae* and *Ectothiorhodospiraceae*, some *Rhodospirillaceae* and some thiobacilli (Dahl and Prange, 2006). For some of the latter, transient intracellular sulfur accumulations has also been reported (Schedel and Trüper, 1980; Hazeu *et al.*, 1988). Here, we report on endosymbiotic bacteria with extracellular sulfur crystals that either originate from intracellular sulfur vesicles or are directly deposited extracellularly.

We suggest that the oxidation of sulfide to elemental sulfur performed by the symbionts of *S. contortum* may serve not only as energy generation and storage for the bacteria themselves, but also provide another benefit for the host in this mutualistic association. Sulfide oxidation to elemental sulfur will reduce the amount of toxic sulfide in the host and thus help the host to deal with exposure to the sulfide that the symbionts require. Such behaviour in which the partner provides goods to the other at no costs, but as an automatic, coincident consequence of a selfish trait is termed by-product benefit and is considered one of the driving forces in the evolution and maintenance of mutualism (West Eberhard, 1975; Connor, 1995; Hauert *et al.*, 2006).

Many animals are capable of detoxifying sulfide without the aid of symbionts. However, none of these animals produce large sulfur crystals such as we detected in *S. contortum*. Sulfide conversion into different non-toxic sulfur compounds, mostly sulfite, sulfate, or thiosulfate, is a widespread phenomenon of free-living animals and of animals associated with thiotrophic bacteria in sulfidic environments. Sulfide oxidation activity has been demonstrated in *Nereis polychaete* worms (Vismann, 1990) and the aposymbiotic host tissue of the clam *Solemya reidi* (Powell and Somero, 1985). The isopode *Saduria entomon* detoxifies sulfide to thiosulfate and sulfite (Vismann, 1991b), the vent crab *Bythograea thermydron* to thiosulfate and sulfate (Vetter *et al.*, 1987). Representatives of platyhelminthes and gastrotrichs produce thiosulfate, but also elemental sulfur as primary end products (Powell *et al.*, 1980).

The sulfur-oxidizing endosymbionts of *S. contortum* exploit the energy contained in sulfide and provide nutrition to the gutless host (Eichinger *et al.*, 2011). Additionally, we propose that when conditions exist that cause internal sulfide levels to exceed the animals ability to supply oxygen to the endosymbionts for oxidation all the way to sulfate, the endosymbionts prevent sulfide accumulation and poisoning by converting sulfide to non-toxic elemental sulfur, stored reversibly in intracellular bacterial vesicles and deposited in large, extracellular crystals. This allows *S. contortum* to inhabit environments with higher sulfide levels than would otherwise be possible. We suggest that the sulfide-oxidizing and -detoxifying function of the symbiont was potentially as important in the evolution of the symbiosis as the more obvious nutritional benefits to the host.

## Supporting Information

Endosymbiont driven sulfur crystal formation.
