# Supplementary material for: Symbiont-driven sulfur crystal formation in a thiotrophic symbiosis from deep-sea hydrocarbon seeps
Source: Environ Microbiol Rep. 2014 Mar 3;6(4):364–72. doi: 10.1111/1758-2229.12149 (PMC4232855; doi:10.1111/1758-2229.12149)
Supplement: Supplementary file 1 — Endosymbiont driven sulfur crystal formation. [file emi40006-0364-SD1.doc]

Supporting information

16S rRNA gene sequencing of the symbiont

For purification of bacterial DNA from trophosomal tissue DNeasy Blood & Tissue Kit (Qiagen, Hilden, Germany) was applied. Bacterial 16S rRNA gene was amplified using 34 PCR cycles with primers 616V and 1492R and an annealing temperature of 50°C. PCR products of the desired size were cloned with TOPO TA cloning kit (Invitrogen Life Technologies, Lofer, Austria). Nucleotide sequences of cloned DNA fragments were determined on an ABI 3130 XL genetic analyzer using the BigDye Terminator kit v3.1 (Applied Biosystems, Austria).

Fluorescence *in situ* hybridizations

Ethanol fixed samples were embedded in LR-White resin after Nussbaumer et al. , semithin-sectioned on a Reichert Ultracut S microtome, mounted on gelatine/chromalaun coated glass slides and dried on a hot plate less than 50°C overnight. Glass slides were subsequently dipped into 50% ethanol, 75% ethanol and 96% ethanol for three minutes each immediately before *in situ* hybridization with oligonucleotide probes. Hybridizations with an incubation time of 4.5h and subsequent staining with DAPI were carried out as described previously . We used universal bacterial probes (EUB338, and GAM42a, ), a symbiont specific probe (Scon-467, ) and a nonsense probe (NON338, ) as a negative control. A 10% formamide concentration gave the best signal for the symbiont specific probe and therefore was applied for all probes.

High-pressure freezing and freeze substitution for LM

Samples fixed in 4% formaldehyde buffered with 0.1 mol l^-1^ phosphate-buffered saline (PBS) were high-pressure frozen using a Leica HPM 100 and stored at -150°C. Freeze-substitution and cryo-embedding in Lowicryl HM20 resin were carried out by using a Leica AFS 2 machine. For freeze-substitution temperature was kept at -90°C for 8 h, raised to -70°C within 2 h, kept at -70°C for 2 h, and raised to -50°C within 4 h. These steps were carried out in 0.1% OsO_4_ in 100% acetone, followed by three washing steps with 100% acetone for 1 h each at -50°C. The samples were infiltrated by a 1:1 followed by a 1:2 100% acetone and resin mixture for 1h each. After infiltration with pure resin for 1 h samples were transferred to gelatine capsules filled with fresh resin and polymerised during a 24 h UV exposure at -50°C. Subsequent hardening of the resin at room temperature with UV light was necessary.

Amann, R.I., Binder, B.J., Olson, R.J., Chisholm, S.W., Devereux, R., and Stahl, D.A. (1990) Combination of 16S rRNA-targeted oligonucleotide probes with flow cytometry for analyzing mixed microbial populations. *Appl Environ Microbiol* **56**: 1919-1925.

Juretschko, S., Timmermann, G., Schmid, M., Schleifer, K.H., Pommerening-Roser, A., Koops, H.P., and Wagner, M. (1998) Combined molecular and conventional analyses of nitrifying bacterium diversity in activated sludge: *Nitrosococcus mobilis* and *Nitrospira*-like bacteria as dominant populations. *Appl Environ Microbiol* **64**: 3042-3051.

Lösekann, T., Robador, A., Niemann, H., Knittel, K., Boetius, A., and Dubilier, N. (2008) Endosymbioses between bacteria and deep-sea siboglinid tubeworms from an artic cold seep (Haakon Mosby Mud Volcano, Barents Sea). *Environ Microbiol* **10**: 3237-3254.

Loy, A., Schulz, C., Lucker, S., Schopfer-Wendels, A., Stoecker, K., Baranyi, C. et al. (2005) 16S rRNA gene-based oligonucleotide microarray for environmental monitoring of the betaproteobacterial order "*Rhodocyclales*". *Appl Environ Microbiol* **71**: 1373-1386.

Manz, W., Amann, R., Ludwig, W., Wagner, M., and Schleifer, K.H. (1992) Phylogenetic oligodeoxynucleotide probes for the major subclasses of proteobacteria : problems and solutions. *Syst Appl Microbiol* **15**: 593-600.

Nussbaumer, A.D., Fisher, C.R., and Bright, M. (2006) Horizontal endosymbiont transmission in hydrothermal vent tubeworms. *Nature* **441**: 345-348.

Wallner, G., Amann, R., and Beisker, W. (1993) Optimizing fluorescent in situ hybridization with rRNA-targeted oligonucleotide probes for flow cytometric identification of microorganisms. *Cytometry* **14**: 136-143.
